# Meta-analysis using Python: a hands-on tutorial

**DOI:** 10.1186/s12874-022-01673-y

**Published:** 2022-07-12

**Authors:** Safoora Masoumi, Saeid Shahraz

**Affiliations:** 1 Pediatric Infectious Diseases Research Center, Mazandaran University of Medical Sciences, Boo- Ali Sina Hospital, Pasdaran Blvd, Sari, Mazandaran 48158 38477 Iran; 2 Institute for Clinical Research and Health Policy Studies, Tufts Medical Center, Boston, USA

**Keywords:** Python, Meta-analysis, PythonMeta, zEpid, Haloperidol, Tutorial

## Abstract

**Background:**

Meta-analysis is a central method for quality evidence generation. In particular, meta-analysis is gaining speedy momentum in the growing world of quantitative information. There are several software applications to process and output expected results. Open-source software applications generating such results are receiving more attention. This paper uses Python’s capabilities to provide applicable instruction to perform a meta-analysis.

**Methods:**

We used the PythonMeta package with several modifications to perform the meta-analysis on an open-access dataset from Cochrane. The analyses were complemented by employing Python’s zEpid package capable of creating forest plots. Also, we developed Python scripts for contour-enhanced funnel plots to assess funnel plots asymmetry. Finally, we ran the analyses in R and STATA to check the cross-validity of the results.

**Results:**

A stepwise instruction on installing the software and packages and performing meta-analysis was provided. We shared the Python codes for meta-analysts to follow and generate the standard outputs. Our results were similar to those yielded by R and STATA.

**Conclusion:**

We successfully produced standard meta-analytic outputs using Python. This programming language has several flexibilities to improve the meta-analysis results even further.

**Supplementary Information:**

The online version contains supplementary material available at 10.1186/s12874-022-01673-y.

## Background

The use of quantitative evidence synthesis methods, i.e., meta-analysis, is rising. The compelling need for applying evidence-based medicine to clinical practice and the generation of an enormous amount of evidence is presumed motivations behind the upward trend in conducting meta-analysis [1, 2]. Cochrane Training, a known public institution aiming to standardize the systematic review and meta-analysis methods in medicine, has developed RevMan to fulfill the growing need for meta-analysis [3]. Several other specialized software applications for meta-analysis exist, e.g., Comprehensive MetaAnalysis [4]. These applications typically offer more or less an inclusive and standard output used by meta-analysts. Generic statistical programs like STATA also provide a full range of typical meta-analysis results [5].

In parallel with commercial programs, the use of open-source applications such as R is also ratcheting up. R provides a host of standard results and graphical displays for meta-analysis [6]. Python is new to the world of meta-analysis. However, given its ease of use and popularity among data scientists, it is not surprising to witness Python’s incremental use for meta-analysis soon. The automatization of systematic reviews by employing natural language processing in Python is getting more recognition [7, 8]. Hence, integrating automated systematic review and meta-analysis in Python can be a promising future endeavor for evidence synthesis as a practical example.

Python program developers have introduced several meta-analysis applications that are at different stages of development-two of them with satisfying features are PythonMeta (PyMeta) [9] and PyMare [10]. However, these applications have been infrequently applied to real-world data. To this date, few researchers have published the capabilities and accuracy of Python-based packages for meta-analysis in peer-reviewed journals. This paper applies Python’s meta-analysis features to a publicly available dataset prepared for this purpose. We aim to explain a stepwise approach to analyzing the data and compare them against R and STATA’s output.

## Methods

### Data

We used the dataset provided by Higgins et al. [11], a subset of data that belongs to the Cochrane study titled “haloperidol versus placebo for schizophrenia” [12]. The dataset comprises 17 different clinical trials to compare haloperidol’s efficacy with placebo [12]. The Cochrane study data is publicly available [11, 13].

### Variables

The following variables and labels (in parenthesis) have been specified for each of these trials: author (author), year of publication (year), haloperidol responders (resp.h), placebo responders (resp. p), haloperidol non-responders (fail.h), and placebo non-responders (fail. p). The dataset also conveys two additional variables, labeled as drop.h, and drop.p, to designate the haloperidol dropouts and placebo arms. PythonMeta to perform meta-analysis needs four input variables haloperidol responders (resp.h), placebo responders (resp. p), and total number in haloperidol (T.h) and total number in Placebo group (T.p). Accordingly, we modified the dataset to facilitate its future use with Python. The modified dataset is available for readers (Additional file 1).

The outcome of interest is the clinical improvement measured as risk ratio (RR), which serves as the selected effect size for the evidence synthesis in this study. RR greater than unity suggests haloperidol’s efficacy against placebo [13].

### Meta-analysis methods

Fixed-effect models assume a fixed effect size across studies. On the other hand, random-effects models allow the effect size to vary from study to study. While understanding the two models’ conceptual differences is crucial for model selection, the discussion is beyond this paper’s scope. For a quick review of the basics of meta-analysis, we highly recommend the paper by Bornstein et al. [14]. Of important note, the analyst needs an adequate level of familiarity with the statistical methods used to estimate these models [15]. In PythonMeta, the default method for the fixed-effect model is Mantel–Haenszel (MH), which can be changed into “Peto” and “IV” for the inverse variance. The package offers a random-effects estimation method to obtain the between-study variance (tau^2^) through the DerSimonian and Laird (DL) method.

### Analysis steps

#### Step 1: installing the program and reading the data

To perform Meta-analysis in Python, PythonMeta (V.1.23) needs to be installed via “pip install PythonMeta” (Reference: On https://pypi.org/project/PythonMeta/ [16]). After installing the package, the Help()function shows help information of PythonMeta. PythonMeta provides Evidence-based medicine (EBM) tasks, such as: Combining effect measures OR (Odds Ratio), RR (Risk Ratio), RD (risk difference) for count data and MD (mean difference), SMD (standardized mean difference)for continuous data; Heterogeneity test(Q/Chi-square test); Subgroup analysis, and plots drawing including forest plot, funnel plot [16]. Pymeta is an online version of the PythonMeta tool (https://www.pymeta.com/) [10].

After preparing the dataset (see the section “variables” above), the dataset sitting in the same file directory as Python scripts can be uploaded directly via readfile (“Haloperidol.text”) [16]. Of note, PythonMeta offers a web-based application, which facilitates direct data entry and provides a few additional analytics [9].

#### Step 2: generating the main results

First, we selected the binary (“CATE” in PythonMeta) outcome and Risk Ratio (“RR”) as the desired effect size. Other options are continuous (“CONT”) for the outcome of interest and Odds Ratio (“OR”) and risk difference (“RD”) for the desired effect size. Second, we preferred to run both fixed-effect and random-effects models. This choice was for demonstration purposes. However, our a priori assumption was compatible with the latter. In the third step, we selected MH (Mantel–Haenszel) to run the fixed-effect and DL (DerSimonian and Laird) to run the random-effects models. Forest plots and funnel plots are the main outputs of this analysis step. One can update the default Python scripts to generate cleaner and more informative visuals [16].

#### Step 3. Assessing the impact of missing data

To understand the impact of missing data, we cleaned the dataset via a simple code available in Additional file 2. After preparing the dataset, the studies with missing and non-missing patients were labeled with “<subgroup>name = Missing” and “<subgroup>name = non-Missing,” and we analyzed them as subgroups. The dataset is available in Additional file 3.

It is common to impute the dataset in several ways to evaluate the impact of completed data on the results. Unlike R, Python meta-analysis packages do not handle an inclusive list of standard missing data imputation methods. Hence, we added a selection of missing data imputation methods after meta-analysis in this paper. The methods are Available Case Study (ACS), Imputed Case Analysis (ICA), and best and worst-case scenarios. ICA-0 is the designation under the assumption that none of the missing participants experience the event. ICA-1 assumes that all of the missing participants experience the event. Also, we used ICA-b for the best-case scenario, assuming all missing participants in the experimental group and none in the control group experienced the event. ICA-w, used for the worst-case scenario, is the reverse of ICA-b [11]. To create a dataset for each method as mentioned above, we used the original dataset of Cochrane with six variables. (resp.h, fail.h, drop.h, resp.p, fail.p,drop.p) (Additional file 4) and wrote code for each method. Next, we ran a separate random-effects model with IV method on each. Using zEpid package, we generated the relevant forest plots [17].

#### Step 4: evaluating the small study effect

Small-study effects occur when small studies, relative to larger ones, demonstrate different, often larger, treatment effects. Funnel plots are a standard way of showing such an effect by measuring their symmetry [15, 18]. In assessing the funnel plots’ asymmetry, several tests such as Egger’s test indicate whether the association between estimated effects and study size is greater than that expected to occur by chance [15, 18]. There are complementary methods to enhance the assessment of small-study effects and conduct sensitivity analysis on the results; however, Python packages do not offer these extended analyses. We perform Egger’s test by applying Statsmodels linear regression.

### Comparison with R and STATA

We used STATA (Release 16. College Station, TX: StataCorp LLC) and R (R Core Team, 2021) for the comparison of the results. Balduzzi et al. [13] and Chaimani et al. [19] used the same dataset we employed in the current study to conduct a meta-analysis. We used the respected STATA and R scripts these authors provided to obtain the results for this comparison.

## Results

Additional file 2 contains the Python scripts to obtain the outputs. To generate the illustrations in this paper, we modified the original Python scripts where needed and added more commands to complete the analysis.

### Fixed-effect and random-effects models

**Fig. 1:**
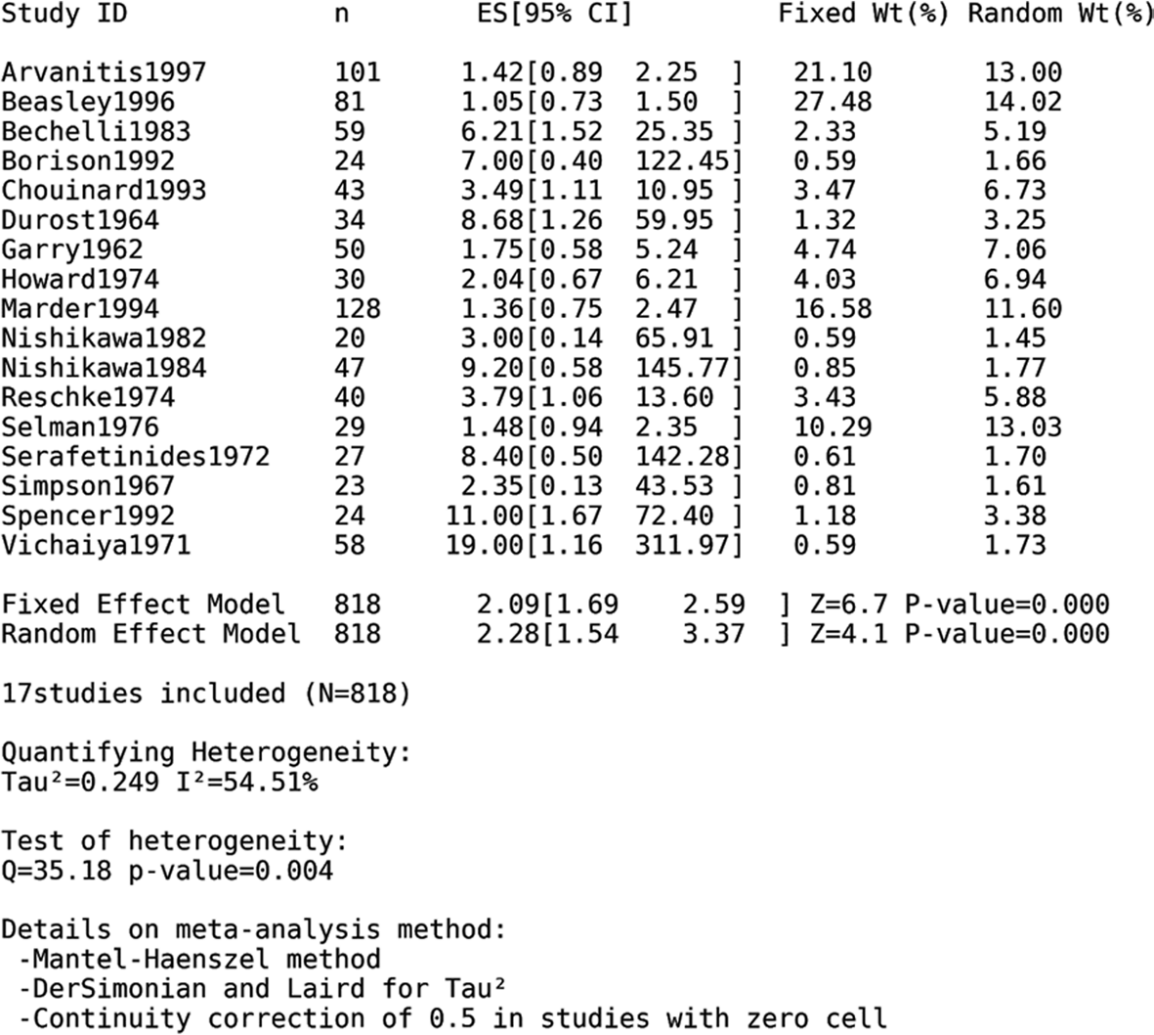
The results of the Fixed and random effect Meta-analysis

**Fig. 2:**
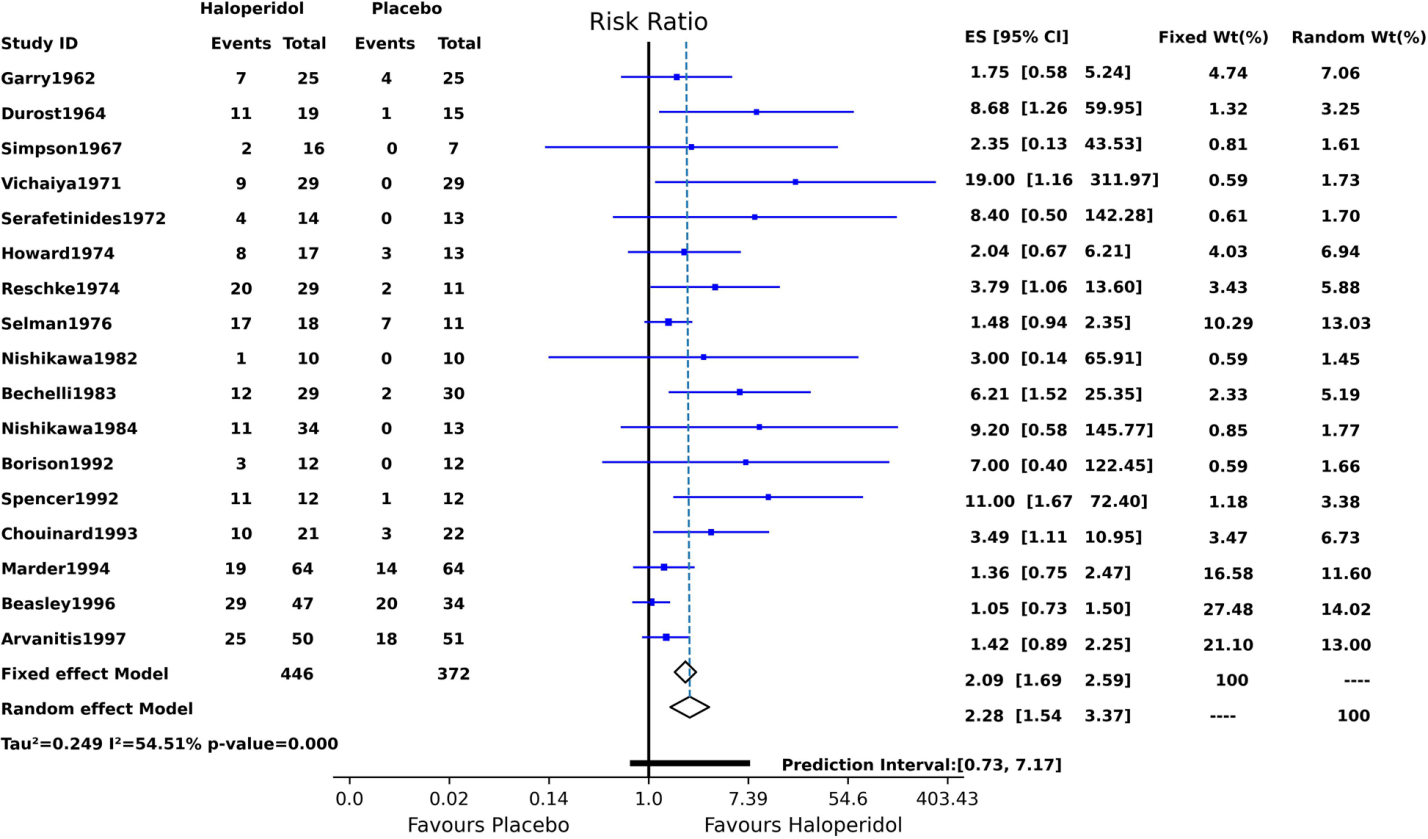
Forest plot showing the results of fixed effect and random effects meta-analysis (ES: effect size)

Figure 1 is the printout display of the PythonMeta function and conveys the essential information about the individual studies, fixed-effect, and random-effects results, heterogeneity, and methods. Figure 1 shows both fixed-effect and random-effects outputs for non-missing cases, with both models indicating a statistically significantly higher haloperidol efficacy than placebo. The overall treatment effect estimated by the fixed-effect model risk ratio was 2.09 (95% CI 1.69,2.59), and the corresponding estimate via the random-effects model was 2.28 (95% CI, 1.54, 3.37). The two diamonds in Fig. 2 represent the overall treatment effects; they do not cross the no-effect vertical bar (RR = 1) and are on the no-effect bar’s right side. The confidence interval for the overall treatment effect using the random-effects model was slightly wider than that of the fixed-effect model. The relatively wide prediction interval (0.73–7.17), taking account of the between-study heterogeneity, crosses the no-effect bar. This finding indicates that future studies may not approve haloperidol’s superior efficacy. Several individual publications showed non-overlapping confidence intervals. This finding and that of the Q test (35.18, *p* value = 0.004) showed heterogeneity in the results. The I^2^ of 54.51% was also an indication of moderate heterogeneity.

### Impact of missing data

**Fig. 3:**
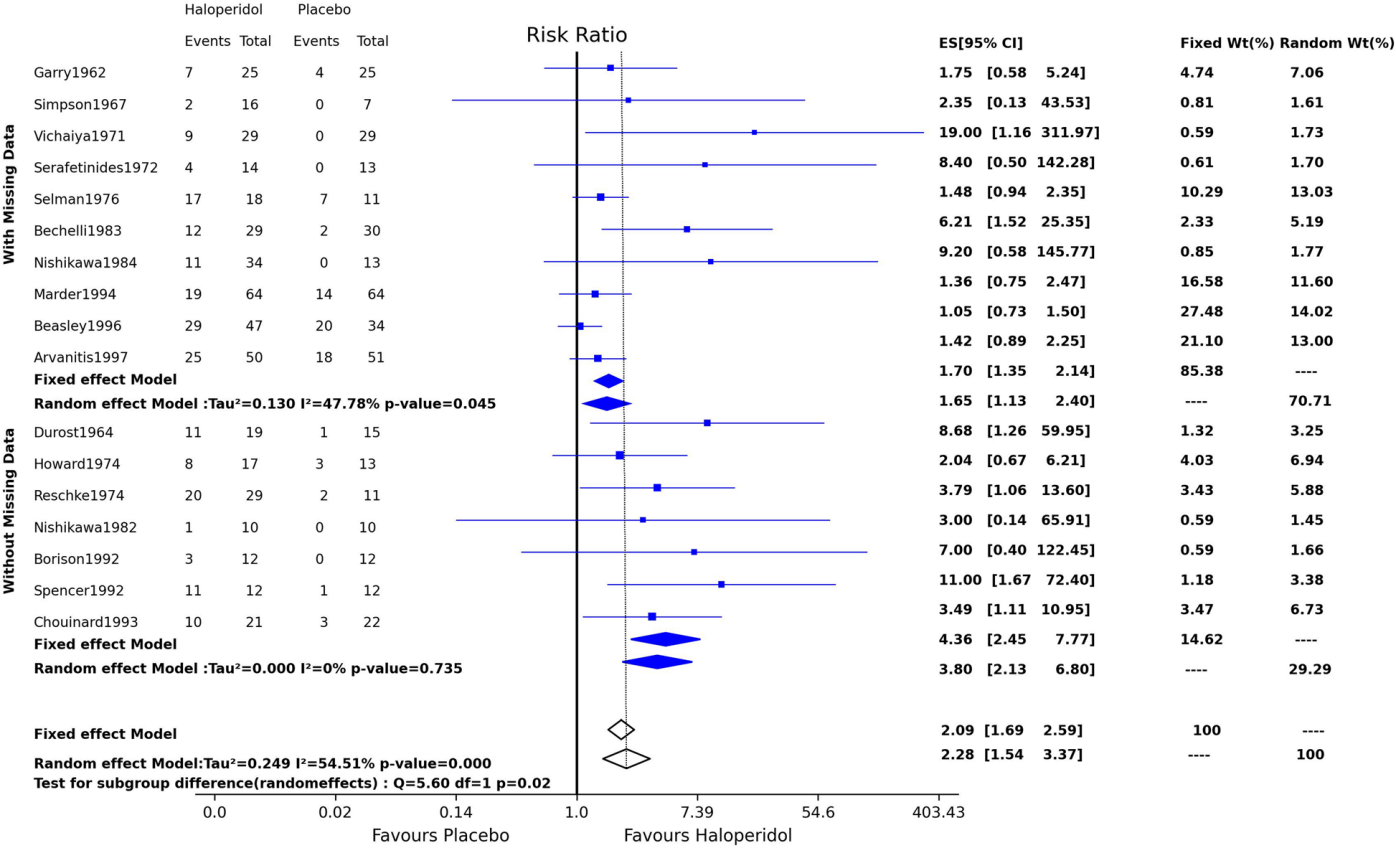
Forest plot showing the subgroup analysis of studies with and without missing data

**Fig. 4:**
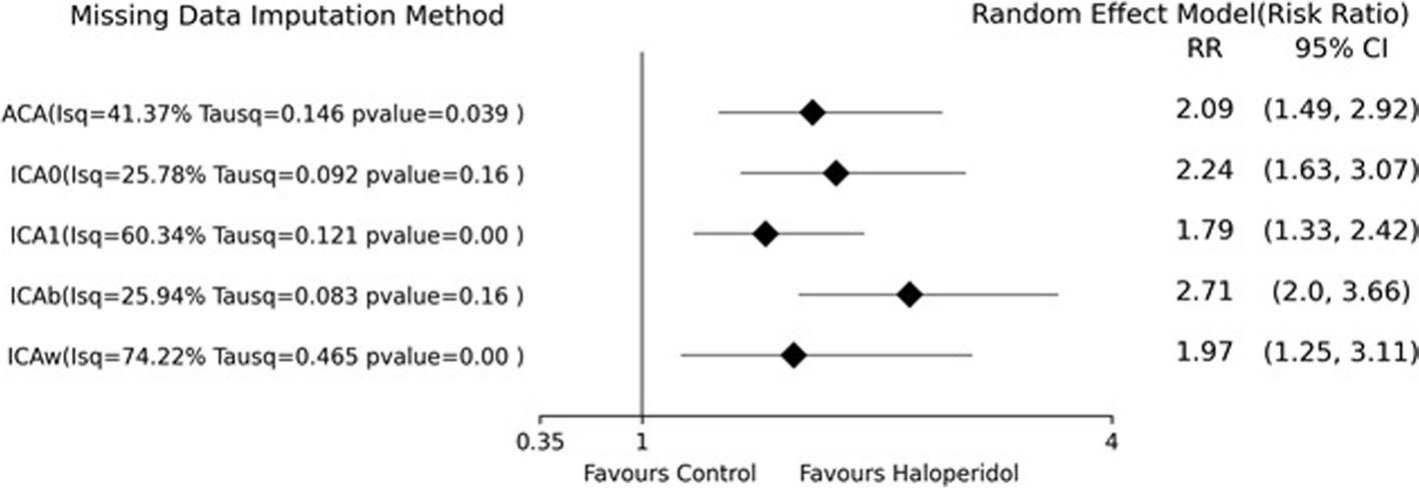
Comparison of summary Risk Ratios (RR) according to different missing data imputation methods

Figure 3 is a forest plot dividing studies with and without missing. The overall treatment effect for both subgroups favors statistically significantly higher haloperidol efficacy than placebo. However, the overall treatment effect for the studies with non-missing data is larger than those with missing data. Several confidence intervals for the subgroup estimates do not include the related overall treatment effect. The chi-square test result under the random-effects model showed a statistically significant difference between the two sub-groups (χ^2^ = 5.60, DF = 1, *p* = 0.02). Figure 4 illustrates the summary results of sensitivity analysis after imputing missing data with five different assumptions about the missing pattern. For example, the risk ratios range between 1.97 and 2.71 for worst and best-case scenarios. Despite different assumptions, the risk ratios and their confidence interval are all on the no-effect bar’s right side and do not cross the bar.

### Assessing small study effect

**Fig. 5:**
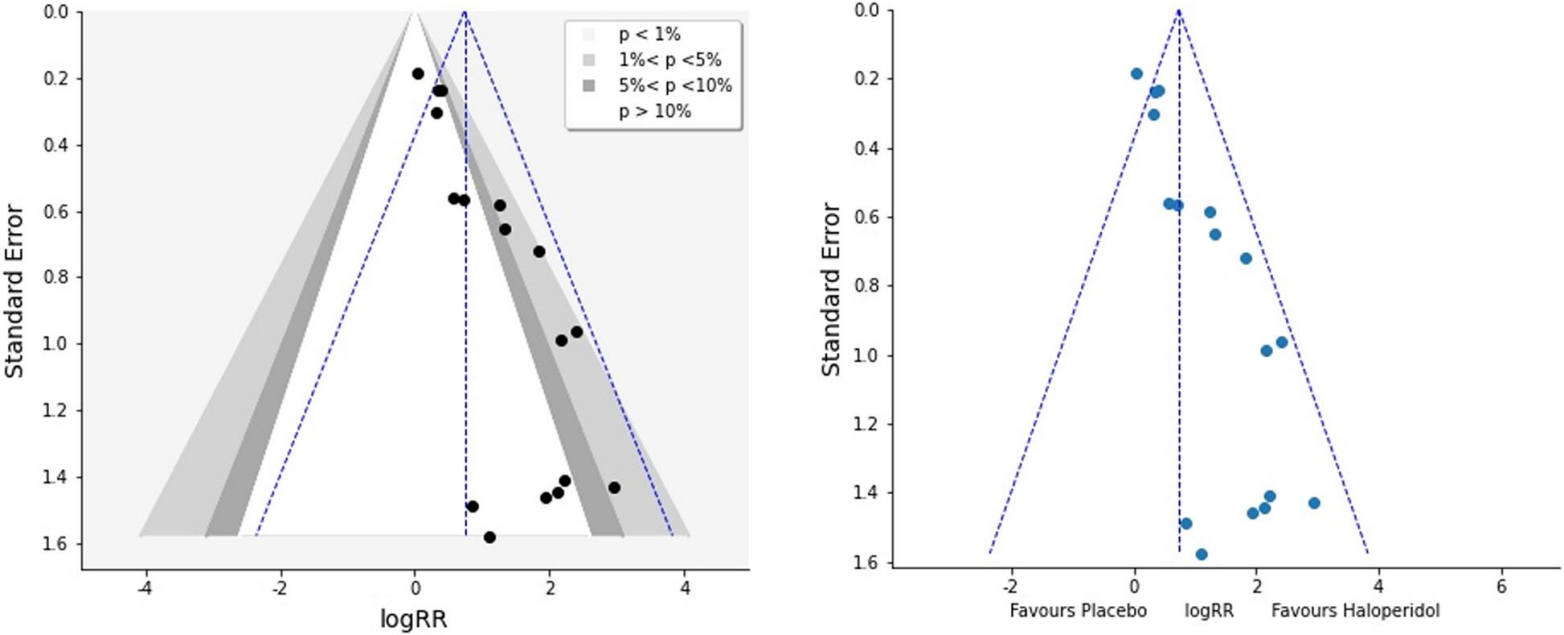
Funnel plot and Contour-enhanced funnel plot to evaluate funnel plot asymmetry. The vertical line corresponds to the estimated summary log (RR) from the fixed effect model, Mantel–Haenszel model method (RR, risk ratio)

The final funnel plot (Fig. 5) is asymmetric. This asymmetry raised the concern of small-study effects. We can see that smaller studies tend to show more efficacy of haloperidol. A contour-enhanced plot is a method to help discern the existence of asymmetry due to a publication bias by demarking the areas of statistical significance for the treatment effect [20]. The contour-enhanced plot shows that small studies present either in with and contoured area. To evaluate the funnel plot asymmetry and small study effect, we performed Egger’s meta-regression test. Examining the result, we can see that the confidence interval of the intercept does not include zero, so we can say that small studies effects are not likely to cause a publication bias. Table 1.

**Table 1:** Egger’s test result for assessing funnel plot symmetry and small study effect

	coef	std err	t	P > |t|	95% CI
Intercept	−0.1463	0.109	−1.342	0.200	−0.379 - 0.086
Bias	1.7894	0.257	6.950	0.000	1.241–2.338

Sensitivity analysis such as trim-fill, yet to be developed in Python, can further help examine the presence of publication bias [21].

### The comparison with R and STATA

Table 2 summarizes the results of the critical meta-analysis parameters across the three different applications. The discrepancies across the three applications are in bold font. Risk ratios obtained using Python were compatible with STATA 100% of the time at the second decimal position. These results were discrepant with those of R in three of seventeen rows. However, the risk ratios were an exact match across rows and columns at the integer levels. The 95% confidence intervals of the risk ratios estimated using Python were equal to those calculated using STATA and R in most cases. The disagreements were notably at the second decimal positions. One can observe the same level of absolute agreement across different applications for the fixed effects, random effects, and summary statistics shown at the bottom of the table. The workflow and computation time between the three software is negligible; the generation of the results takes no more than a few seconds.

**Table 2:** Risk Ratio (RR with 95% Confidence Intervals, Fixed Effects (FE), Random Effects (RE)) and the meta-analysis summary statistics using Python, STATA, and R

IV, Random, Fixed, DL	Python			STATA			R		
	Weight			Weight			Weight		
**Author year**	**RR (95%CI)**	**FE**	**RE**	**RR (95%CI)**	**FE**	**RE**	**RR (95%CI)**	**FE**	**RE**
Arvanitis1997	1.42(0.89–2.25)	18.86	**14.66**	1.42(0.89–2.25)	18.86	**14.66**	1.42 (0.89–2.25)	18.86	**14.67**
Beasley1996	1.05(0.73–1.50)	31.22	**16.46**	1.05(0.73–1.50)	31.22	**16.46**	1.05 (0.73–1.50)	31.22	**16.48**
Bechelli1983	6.21(1.52–25.35)	2.05	4.48	6.21(1.52–25.35)	2.05	4.48	6.21 (1.52–25.35)	2.05	4.48
Borison1992	7.00(0.40–**122.45**)	**0.49**	1.30	7.00(0.40–**122.44**)	**0.49**	1.30	7.00 (0.40–**121.94**)	**0.50**	1.30
Chouinard1993	3.49(1.11–**10.95**)	3.10	**6.10**	3.5(1.11–**10.96**)	3.10	**6.10**	3.49 (1.11–**10.95)**	3.10	**6.09**
Durost1964	8.68(1.26–59.95)	1.09	2.65	8.68(1.26–59.95)	1.09	2.65	8.68 (1.26–59.95)	1.09	2.65
Garry1962	1.75(**0.58**–5.24)	3.37	**6.46**	1.75(**0.59**–5.24)	3.37	**6.46**	1.75 (**0.58–**5.24)	3.37	**6.45**
Howard1974	2.04(0.67–6.21)	3.27	**6.33**	2.04(0.67–6.21)	3.27	**6.33**	2.04 (0.67–6.21)	3.27	**6.32**
Marder1994	1.36(0.75–2.47)	11.37	**12.40**	1.36(0.75–2.47)	11.37	**12.40**	1.36 (0.75–2.47)	11.37	**12.41**
Nishikawa1982	3.00(0.14–**65.91**)	0.42	1.13	3.00(0.14–**65.90**)	0.42	1.13	3.00 (0.14–**65.55**)	0.43	1.13
Nishikawa1984	**9.20(0.58**–**145.77**)	0.53	1.39	**9.20**(**0.58**–**145.76**)	0.53	1.39	**9.00** (**0.57**–**142.29**)	0.53	1.39
Reschke1974	3.79(1.06–13.60)	2.48	**5.20**	3.79(1.06–13.60)	2.48	**5.20**	3.79 (1.06–13.60)	2.48	5.19
Selman1976	1.48(0.94–2.35)	19.11	**14.71**	1.48(0.94–2.35)	19.11	**14.71**	1.48 (0.94–2.35)	19.11	**14.72**
Serafetinides1972	**8.40**(0.50–**142.28**)	0.51	1.33	**8.40**(0.50–**142.28**)	0.51	1.33	**8.38** (0.50–**141.44**)	0.51	1.33
Simpson1967	**2.35(0.13–43.53)**	0.48	1.26	**2.35(0.13–43.53)**	0.48	1.26	**2.27 (0.12–41.77)**	0.48	1.26
Spencer1992	11.00(1.67–72.40)	1.14	2.77	11.00(1.67–72.40)	1.14	2.77	11.00 (1.67–72.40)	1.14	2.77
Vichaiya1971	19.00**(**1.16**–311.97)**	0.52	1.36	19.00**(**1.16**–311.96)**	0.52	1.36	19.00 **(**1.16**–311.71)**	0.52	1.36
Total	Fix Rand			Fix Rand			Fix Rand		
Random	**2.09**(1.49–2.92)			**2.09**(1.49–2.92)			**2.08**(1.49–2.92)		
Fixed	1.57(1.28–1.92)			1.57(1.28–1.92)			1.57(1.28–1.92)		
Tau^2^	0.146			0.146			0.146		
I^2^	**41.37**			**41.37**			**41.27**		
Q	**27.29**			**27.29**			**27.24**		
P	0.03			0.03			0.03		
Z	4.37–**4.27**			4.37–**4.27**			4.37**–4.26**		

## Discussion

Meta-analysis and systematic reviews are improving tools for evidence generation and synthesis [1, 2]. An automated systematic review is also a growing method that uses NLP algorithms [7, 8]. Hence, one can anticipate the increasing use of omnibus data handling and data analysis packages like Python for evidence generation and meta-analytic analysis at the same time. To introduce Python’s capabilities and show the accuracy of the meta-analysis estimates, we used the PythonMeta package to run the meta-analysis. We selected PythonMeta over its competitor algorithms such as PyMAre to fit our purpose. The strength of PyhonMeta lies in its web-based algorithm that eases its application and diverse options to generate standard outputs for scientific publications [9, 10, 16].

Using a binary outcome from a publicly available dataset and employing zEpid package to create a forest plot for the missing data imputations, we could demonstrate the accuracy of the results. Python, STATA, and R generated comparable results for the standard parameters. Evaluation of funnel plot asymmetry combined with contour enhanced funnel plot revealed a small study effect that publication bias could not entirely explain. While the Python package lacked sensitivity analysis tests, we showed a non-significant treatment effect using R and STATA standard packages for meta-analysis. Clinical heterogeneity is another unchecked source of variability that might explain the treatment effect diversification [22].

By analyzing subgroups with and without missing data, we indicated a more significant haloperidol effect in the subgroup without missingness than those with missing data. Unfortunately, the Python package lacked the capability of quantifying the between-group heterogeneity. We could, however, assess this heterogeneity by visually attending to the overlapping confidence intervals in the summary estimates [23].

We identified several gaps concerning Python meta-analytic capabilities.

- Algorithms for sensitivity analysis
- Missing data imputations
- Regression analysis
- Counter-enhanced funnel plots
- Subtle but indispensable details such as between-group heterogeneity quantifications, the prediction interval

We tried to address some of these gaps by modifying the existing Python macros for meta-analysis. However, these items provide a roadmap for future meta-analytic improvements in Python.

## Conclusion

In this paper, we introduce Python as a tool for meta-analysis. We took advantage of Python-based packages written for meta-analysis, modified them, and generated standard meta-analytic results. The comparison of these results with STATA and R’s outputs supports the accuracy of our algorithms.

## Supplementary Information

**Additional file 1:** Haloperidol.txt

**Additional file 2:** Code.docx

**Additional file 3:** Subgroup.txt

**Additional file 4:** Cochrane.csv

**Additional file 5:** 

**Additional file 6:** 

## Data Availability

“The dataset(s) supporting the conclusions of this article is (are) included within the article (and its additional file(s)). Modified sample codes and new Python scripts are available in Additional files. The PythonMeta package can be installed via *pip install PythonMeta.* The source code is available at https://pypi.org/project/PythonMeta/#files. Archived versions are available from https://pypi.org/project/PythonMeta/#history. The dataset used in this article is accessible via below links: Cochrane dataset of haloperidol versus placebo for schizophrenia. https://www.cochranelibrary.com/cdsr/doi/10.1002/14651858.CD003082.pub3/full https://www.ncbi.nlm.nih.gov/pmc/articles/PMC2602608/

## Abbreviations

RR: Risk Ratio
OR: Odds Ratio
RD: Risk Difference
SE: Standard Error
MH: Mantel–Haenszel
IV: Inverse Variance
DL: DerSimonian and Laird
ACS: Available Case Study
ICA: Imputed Case Analysis
NLP: Natural Language Processing
