# Supplementary material for: Meta-analysis using Python: a hands-on tutorial
Source: BMC Med Res Methodol. 2022 Jul 12;22:193. doi: 10.1186/s12874-022-01673-y (PMC9275021; doi:10.1186/s12874-022-01673-y)
Supplement: Supplementary file 2 — Additional file 2: Code.docx [file 12874_2022_1673_MOESM2_ESM.docx]

**The following code consists of 11 sets of code, for figures and table. If you put the dataset in the same folder as the code, you can read it directly, otherwise you should use the address.**

**The package has Funnel plot, but we wrote the code as well.**

**For meta-analysis, you should always choose the data type first**

**Table 1: Egger`s test result for assessing funnel plot symmetry and small study effect**

**#Eggers`s test: is obtained by regressing the standardized effect size on the inverse of the standard error (the precision)**

**# We use this test to assess the funnel plot asymmetry**

import PythonMeta as PMA

import math

import numpy as np

import statsmodels.api as sm

def SEeff(results): #effect size

SND=[]

for i in range(1, len(results)):

SEF =math.log(results[i][1])/results[i][6]

SND.append(SEF)

return(SND)

def RevSE(results): #precision

Se=[]

for i in range(1, len(results)):

rese =1/results[i][6]

Se.append(rese)

return(Se)

def main(setting):

d = PMA.Data() # Load Data class

m = PMA.Meta() # Load Meta class

d.datatype = setting["datatype"]

studies = d.getdata(d.readfile("Haloperidol.txt"))

m.datatype = d.datatype

m.models = setting["models"]

m.algorithm = setting["algorithm"]

m.effect = setting["effect"]

results = m.meta(studies)

x = np.array(RevSE(results))

y = np.array(SEeff(results))

x = sm.add_constant(x)

est = sm.OLS(y, x).fit()

print(est.summary())

if __name__ == '__main__':

settings = {"datatype": "CATE",

"models": "random",

"algorithm": "iv",

"effect": "RR"}

main(settings)

**Figure 1:**  **The results of the Fixed and random effect Meta-analysis**

**#This is the code to generate Figure1. We modified the package code to obtain more inclusive figure.**

**#We combine fixed effect and random effect in one figure and add details on the bottom of the figure**

#This is the code to generate Figure1. We modified the package code to obtain more inclusive figure.

#We combine fixed effect and random effect in one figure and add details on the bottom of the figure

import PythonMeta as PMA

import matplotlib.pyplot as plt

import math

from matplotlib.lines import Line2D

def showresults(fixed_results, random_results):

text = "{0:<20} {1:<8} {2:18} {3:10} {4}"\

.format("Study ID", "n", "ES[95% CI]","Fixed Wt(%)", "Random Wt(%)\n\n")

for i in range(1, len(fixed_results)):

NewId=(fixed_results[i][0]).replace("<sub>","")

if (fixed_results[i][0]).startswith("<sub>"):

Id= NewId

else:

Id=fixed_results[i][0]

text += "{0:<20} {1:<6} {2:5.2f}[{3:<6.2f}{4:<6.2f}] "\

"{5:<11.2f} {6:<15.2f} \n".format( # for each study

Id, # study ID

fixed_results[i][5], # total num

fixed_results[i][1], # effect size

fixed_results[i][3], # lower of CI

fixed_results[i][4], # higher of CI

100*(fixed_results[i][2]/fixed_results[0][2]), # Fixed weight

100*(random_results[i][2]/random_results[0][2]))# Random weight

text += "\n{0:<20} {1:<7} {2:5.2f}[{3:<6.2f} {4:<6.2f}] Z={5:<.3} P-value={6:<.20} \n".format( # for total effect

"Fixed Effect Model", # total effect size model name

fixed_results[0][5], # total N (all studies)

fixed_results[0][1], # total effect size

fixed_results[0][3], # total lower CI

fixed_results[0][4], # total higher CI

fixed_results[0][10],

fixed_results[0][11]

)

text += "{0:<20} {1:<7} {2:5.2f}[{3:<6.2f} {4:<6.2f}] Z={5:<.3} P-value={6:<.20} \n".format( # for total effect

"Random Effect Model", # total effect size model name

random_results[0][5], # total N (all studies)

random_results[0][1], # total effect size

random_results[0][3], # total lower CI

random_results[0][4], # total higher CI

random_results[0][10],

random_results[0][11],

)

text += "\n{0:<d}studies included (N={1:<d})\n".format( len(fixed_results) - 1, fixed_results[0][5])

text += "\nQuantifying Heterogeneity: \n" "Tau\u00b2={0:.3f} I\u00b2={1:s}\n".format(

random_results[0][12],

str(round(random_results[0][9], 2)) + "%")

text += "\nTest of heterogeneity:\n" "Q={0:.2f} p-value={1:s} \n".format(

fixed_results[0][7], # Q test value

fixed_results[0][8], # p value for Q test

)

text+= "\n{0:s}\n {1:s}\n {2:s}\n {3:s}".format("Details on meta-analysis method:",

"-Mantel-Haenszel method",

"-DerSimonian and Laird for Tau\u00b2",

"-Continuity correction of 0.5 in studies with zero cell")

return text

def main(setting):

d = PMA.Data() # Load Data class

m = PMA.Meta() # Load Meta class

d.datatype = setting["datatype"] # set data type, 'CATE' for binary data or 'CONT' for continuous data

studies = d.getdata(d.readfile("Haloperidol.txt")) # get data from a data file

m.subgroup = d.subgroup

m.datatype = d.datatype # set data type for meta-analysis calculating

m.models = setting["models"][0] # set effect models: 'Fixed'

m.algorithm = setting["algorithm"] # set algorithm, based on datatype and effect size

m.effect = setting["effect"] # set effect size:RR/OR/RD for binary data; SMD/MD for continuous data

results = m.meta(studies) # performing the analysis

m.models = setting["models"][1] #set effect models: Random"

res_random = m.meta(studies)

print(showresults(results, res_random))

#plt.rc('figure', figsize=(8, 8))

#plt.text(0.01, 0.02, str((showresults(results, res_random))), {'fontsize':14},family="open-Sans", fontproperties = 'monospace')

#plt.axis('off')

#plt.savefig('Figure1.png',dpi=400,bbox_inches="tight",orientation="landscape")

if __name__ == '__main__':

settings = {"datatype": "CATE", # for CATEgorical/count/binary/dichotomous data

"models": ["Fixed", "Random"], # models: Fixed or Random

"algorithm": "MH", # algorithm: MH, Peto or IV

"effect": "RR"} # effect size: RR, OR, RD

main(settings)

**#Create datasets for missing and without missing studies**

import numpy as np

import pandas as pd

df=pd.read_csv("Cochrane.csv")

withmiss=df.loc[(df["drop.h"]!=0) | (df["drop.p"] !=0)]

withmiss.to_csv("withMiss.csv",index = False)

Withoutmiss = df.loc[(df["drop.h"] == 0) & (df["drop.p"] == 0)]

Withoutmiss.to_csv("Withoutmiss.csv",index = False)

**Figure 2. Forest plot showing the results of fixed effect and random effects meta-analysis (ES: effect size)**

**#To create the forest plot, we modify the package source code. We included the prediction interval in the plot and**

**#Combined random and fixed effect meta-analysis results.**

import PythonMeta as PMA

import matplotlib.pyplot as plt

import scipy.stats

import math

from pylab import gca

from matplotlib.lines import Line2D

def showstudy(studies):

text = "{0:22}{1:20}\n".format( "Haloperidol ", " Placebo")

text += "{0:80}{1:10}{2:10}{3:10}{4:10}\n".format(" "," Events","Total","Events","Total")

hal=0

pla=0

for i in range(len(studies)):

text += "{0:10}{1:13}{2:10}{3:10}\n".format(

str(studies[i][0]), #event num of group1

str(studies[i][1]), #total num of group1

str(studies[i][2]), #event num of group2

str(studies[i][3]),) #total num of group2

text += "{0:25}{1:10}".format("446","372")

return text

def studyID(studies,random_results):

text="{0:23}\n".format( "Study ID")

for i in range(len(studies)):

text += "{0:10}\n".format(studies[i][4]) #study ID

text+="Fixed effect Model\n""Random effect Model\n""Tau\u00b2={0:.3f} I\u00b2={1:s} p-value={2:s}".format(

random_results[0][12],

str(round(random_results[0][9], 2)) + "%",

random_results[0][11]

)

return text

#Fixed and randome effect weights

def descripweight(fixed_results,random_results):

text = "{0:<16} {1:<}".format(" Fixed Wt(%)" ,"Random Wt(%)\n")

for i in range(1, len(fixed_results)):

text +="{0:15.2f} {1:15.2f}\n".format(

100*(fixed_results[i][2]/fixed_results[0][2]), # Fixed weight

100*(random_results[i][2]/random_results[0][2])) # Random weight

return text

#Show effect size and 95%confidence interval

def descrip(fixed_results,random_results):

text = "{}".format(" ES [95% CI]\n")

for i in range(1, len(fixed_results)):

text +="{:8.2f} [{:.2f} {:.2f}]\n" .format(

fixed_results[i][1], # effect size

fixed_results[i][3], # lower of CI

fixed_results[i][4],) # higher of CI

text += " {0:5.2f} [{1:.2f} {2:6.2f}]{3:>18} {4:>20}\n {5:5.2f} [{6:.2f} {7:6.2f}]{8:>18} {9:>20}".format(

# for total effect

fixed_results[0][1], # total effect size

fixed_results[0][3], # total lower CI

fixed_results[0][4], # total higher CI

"100",

"----",

random_results[0][1], # total effect size

random_results[0][3], # total lower CI

random_results[0][4], # total higher CI

"----",

"100",

)

return text

#calculating prediction intervals ,it is not included in the package

def tausq(random_results): #Tausquared

tausq=(random_results[0][12])

return tausq

def weight(rults): #weight

weight=[]

for i in range(1,len(rults)):

W=rults[i][2]

weight.append(W)

print(weight)

def totalweight(rults): #total weight

TW=rults[0][2]

return TW

def Yi(rults): #log effect size

logES=[]

for i in range(1,len(rults)):

Yi=math.log(rults[i][1])

logES.append(Yi)

print(logES)

def VM(rults):

VM=1/rults[0][2]

return VM

def totalWY(rults): #total weight*log effect size

totalWY=0

for i in range(1,len(rults)):

WY=(rults[i][2])*(math.log(rults[i][1]))

totalWY+=WY

return totalWY

def M(rults): #summary mean

M=totalWY(rults)/totalweight(rults)

return M

def PI(rults):

K=(len(rults)-1)

df=K-2

t=scipy.stats.t.ppf(q=1-.05/2,df=df)

MR=M(rults)

Tausq=tausq(rults)

VMR=VM(rults)

UpperPI=math.exp(MR+t*(math.sqrt(Tausq+VMR)))

LowerPI=math.exp(MR-t*(math.sqrt(Tausq+VMR)))

return ("Prediction Interval:"+ str([float("{:.2f}".format(LowerPI)),float("{:.2f}".format(UpperPI))]))

def main(setting):

d = PMA.Data() # Load Data class

m = PMA.Meta() # Load Meta class

f = PMA.Fig() # Load Fig class

#if you put the dataset in the same folder as the code, you can run it directly, else you should put the address

import PythonMeta as PMA

import matplotlib.pyplot as plt

import scipy.stats

import math

from pylab import gca

from matplotlib.lines import Line2D

def showstudy(studies):

text = "{0:22}{1:20}\n".format( "Haloperidol ", " Placebo")

text += "{0:80}{1:10}{2:10}{3:10}{4:10}\n".format(" "," Events","Total","Events","Total")

hal=0

pla=0

for i in range(len(studies)):

text += "{0:10}{1:13}{2:10}{3:10}\n".format(

str(studies[i][0]), #event num of group1

str(studies[i][1]), #total num of group1

str(studies[i][2]), #event num of group2

str(studies[i][3]),) #total num of group2

text += "{0:25}{1:10}".format("446","372")

return text

def studyID(studies,random_results):

text="{0:23}\n".format( "Study ID")

for i in range(len(studies)):

text += "{0:10}\n".format(studies[i][4]) #study ID

text+="Fixed effect Model\n""Random effect Model\n""Tau\u00b2={0:.3f} I\u00b2={1:s} p-value={2:s}".format(

random_results[0][12],

str(round(random_results[0][9], 2)) + "%",

random_results[0][11]

)

return text

#Fixed and randome effect weights

def descripweight(fixed_results,random_results):

text = "{0:<16} {1:<}".format(" Fixed Wt(%)" ,"Random Wt(%)\n")

for i in range(1, len(fixed_results)):

text +="{0:15.2f} {1:15.2f}\n".format(

100*(fixed_results[i][2]/fixed_results[0][2]), # Fixed weight

100*(random_results[i][2]/random_results[0][2])) # Random weight

return text

#Show effect size and 95%confidence interval

def descrip(fixed_results,random_results):

text = "{}".format(" ES [95% CI]\n")

for i in range(1, len(fixed_results)):

text +="{:8.2f} [{:.2f} {:.2f}]\n" .format(

fixed_results[i][1], # effect size

fixed_results[i][3], # lower of CI

fixed_results[i][4],) # higher of CI

text += " {0:5.2f} [{1:.2f} {2:6.2f}]{3:>18} {4:>20}\n {5:5.2f} [{6:.2f} {7:6.2f}]{8:>18} {9:>20}".format(

# for total effect

fixed_results[0][1], # total effect size

fixed_results[0][3], # total lower CI

fixed_results[0][4], # total higher CI

"100",

"----",

random_results[0][1], # total effect size

random_results[0][3], # total lower CI

random_results[0][4], # total higher CI

"----",

"100",

)

return text

#calculating prediction intervals ,it is not included in the package

def tausq(random_results): #Tausquared

tausq=(random_results[0][12])

return tausq

def weight(rults): #weight

weight=[]

for i in range(1,len(rults)):

W=rults[i][2]

weight.append(W)

print(weight)

def totalweight(rults): #total weight

TW=rults[0][2]

return TW

def Yi(rults): #log effect size

logES=[]

for i in range(1,len(rults)):

Yi=math.log(rults[i][1])

logES.append(Yi)

print(logES)

def VM(rults):

VM=1/rults[0][2]

return VM

def totalWY(rults): #total weight*log effect size

totalWY=0

for i in range(1,len(rults)):

WY=(rults[i][2])*(math.log(rults[i][1]))

totalWY+=WY

return totalWY

def M(rults): #summary mean

M=totalWY(rults)/totalweight(rults)

return M

def PI(rults):

K=(len(rults)-1)

df=K-2

t=scipy.stats.t.ppf(q=1-.05/2,df=df)

MR=M(rults)

Tausq=tausq(rults)

VMR=VM(rults)

UpperPI=math.exp(MR+t*(math.sqrt(Tausq+VMR)))

LowerPI=math.exp(MR-t*(math.sqrt(Tausq+VMR)))

return ("Prediction Interval:"+ str([float("{:.2f}".format(LowerPI)),float("{:.2f}".format(UpperPI))]))

def main(setting):

d = PMA.Data() # Load Data class

m = PMA.Meta() # Load Meta class

f = PMA.Fig() # Load Fig class

d.datatype = setting["datatype"]

studies = d.getdata(d.readfile("Haloperidol.txt"))

m.subgroup = d.subgroup

m.datatype = d.datatype # set data type for meta-analysis calculating

m.models = setting["models"][0] # set effect models: 'Fixed'

m.algorithm = setting["algorithm"] # set algorithm, based on datatype and effect size

m.effect = setting["effect"] # set effect size:RR/OR/RD for binary data; SMD/MD for continuous data

results = m.meta(studies) # performing the analysis

m.models = setting["models"][1] #set effect models: Random"

res_random = m.meta(studies)

f.forest(res_random) #show forest plot

plt.text(6,1.5, descrip(results,res_random),fontsize=8, style='normal', ha="left",

va='bottom',multialignment="left",linespacing=2.55,weight="bold")

plt.text(10,2.5,descripweight(results,res_random),fontsize=8, style='normal', ha="left",

va='bottom',multialignment="left",linespacing=2.55,weight="bold")

plt.text(8.3,0.1, PI(res_random),fontsize=8, style='normal', ha="right",

va='bottom', multialignment="left",linespacing=2.5,weight="bold")

plt.plot((math.log(res_random[0][1]),0),((res_random[0][1]),(len(res_random)+200)),linestyle="--", lw=1)

plt.yticks([])

plt.text(-13,0.8,studyID(studies,res_random),multialignment="left",fontsize=8,weight="bold",linespacing=2.5)

plt.text(-18,2.8,showstudy(studies),multialignment="right",fontsize=8,weight="bold",linespacing=2.5)

plt.title("Risk Ratio",loc="center")

plt.xlabel("{:<38}{:<s}".format(" Favours Placebo","Favours Haloperidol"),fontsize=10)

#draw PI

K=(len(res_random)-1)

df=K-2

t=scipy.stats.t.ppf(q=1-.05/2,df=df)

UpperPI=(M(res_random)+t*(math.sqrt(tausq(res_random)+VM(res_random))))

LowerPI=(M(res_random)-t*(math.sqrt(tausq(res_random)+VM(res_random))))

plt.plot((LowerPI,UpperPI),(0.4,0.4),color="black",lw=3)

#the total effect size diamond for fixed effect model

x=[math.log(results[0][1]),

math.log(results[0][3]),

math.log(results[0][1]),

math.log(results[0][4]),

math.log(results[0][1])]

y=[3.3,3,2.7,3,3.3]

if (results[0][9]<50) :

plt.fill(x,y,color="black", lw=1) #filled: I2<50

else :

plt.plot(x,y, 'black', lw=1) #empty: I2>50

ax=plt.gca()

ax.spines['top'].set_visible(False)

plt.savefig("Forest plot.jpg", dpi=500,bbox_inches="tight",orientation="landscape")

if __name__ == '__main__':

settings = {"datatype": "CATE", # for CATEgorical/count/binary/dichotomous data

"models": ["Fixed", "Random"], # models: Fixed or Random

"algorithm": "MH", # algorithm: MH, Peto or IV

"effect": "RR"} # effect size: RR, OR, RD

main(settings)

**Figure 3. Forest plot showing the subgroup analysis of studies with and without missing data**

**#We change the code considerably to obtain subgroup differences and create a figure with full explanation of studies and each group between-study variances.**

#We change the code considerably to obtain subgroup differences and create a figure with full explanation of studies

#and each group between-study varinces.

import PythonMeta as PMA

import matplotlib.pyplot as plt

import matplotlib

import scipy.stats

import math

from pylab import gca

def showithmiss(studies): #studies with missing data

text = "{0:18}{1:16}\n".format( "Haloperidol ", " Placebo")

text += "{0:<8}{1:<10}{2:<10}{3:10}{4}\n".format("Events","Total","Events","Total"," ")

for i in range(len(studies)):

text += "{0:12}{1:13}{2:^5}{3:>8}\n".format(

str(studies[i][0]), #event num of group1

str(studies[i][1]), #total num of group1

str(studies[i][2]), #event num of group2

str(studies[i][3])) #total num of group2

return text

def showithoutmiss(studies): #studies without missing data

text = "{}\n".format("")

for i in range(len(studies)):

text += "{0:12}{1:13}{2:^5}{3:>8}\n".format(

str(studies[i][0]), #event num of group1

str(studies[i][1]), #total num of group1

str(studies[i][2]), #event num of group2

str(studies[i][3])) #total num of group2

return text

def ID(studies): #studies` names

text = "{0:18}\n".format( " ")

for i in range(len(studies)):

text += "{0}\n".format(

str(studies[i][4]))

return text

def rultswithmiss(fixed_results, random_results): #between-study variance for studies with missing data

text = "{0}\n".format("\n")

for i in range(1, len(fixed_results)):

if (fixed_results[i][0]).startswith("<sub>WithMissingdata"):

text+="{}".format("Fixed effect Model\n")

text+="Random effect Model :Tau\u00b2={0:.3f} I\u00b2={1:s} p-value={2:s}\n".format(

random_results[i][12],

str(round(random_results[i][9], 2)) + "%",

random_results[i][8],)

return text

def rultswithoutmiss(fixed_results, random_results): #between-study variance studies without missing data

text = "{0}\n".format("\n")

for i in range(1, len(fixed_results)):

if (fixed_results[i][0]).startswith("<sub>WithoutMissingdata"):

text+="{}".format("Fixed effect Model\n")

text+="Random effect Model :Tau\u00b2={0:.3f} I\u00b2={1:s} p-value={2:s}\n".format(

random_results[i][12],

str(round(random_results[i][9], 2)) + "%",

random_results[i][8],)

return text

def descrip(fixed_results,random_results): #show total results

text = "{}".format("")

for i in range(1, len(fixed_results)):

text += " {0:5.2f} [{1:.2f} {2:6.2f}] {3:>25} {4:>20}\n {5:5.2f} [{6:.2f} {7:6.2f}] {8:>25} {9:>20}".format(

fixed_results[0][1], # total effect size

fixed_results[0][3], # total lower CI

fixed_results[0][4], # total higher CI

"100",

"----",

random_results[0][1], # total effect size

random_results[0][3], # total lower CI

random_results[0][4], # total higher CI

"----",

"100")

return text

def CI(fixed_results,random_results): #95% confidence interval

text = "{0}".format("ES[95% CI]\n")

for i in range(1, len(fixed_results)):

if (fixed_results[i][0]).startswith("<sub>"):

text += "{0:<6.2f} [{1:3.2f} {2:6.2f}]\n".format(

fixed_results[i][1], # total effect size

fixed_results[i][3], # total lower CI

fixed_results[i][4], # total higher CI

)

text += "{0:<6.2f} [{1:3.2f} {2:6.2f}]\n".format(

random_results[i][1], # total effect size

random_results[i][3], # total lower CI

random_results[i][4], # total higher CI

)

continue

text += "{0:<6.2f} [{1:.2f}{2:8.2f}]\n".format(

fixed_results[i][1], # effect size

fixed_results[i][3], # lower of CI

fixed_results[i][4],

)

return text

def showeight(fixed_results,random_results): #weights

text = "{0:10} {1}".format("Fixed Wt(%)", "Random Wt(%)\n")

for i in range(1, len(fixed_results)):

if (fixed_results[i][0]).startswith("<sub>"):

text += "{0:<17.2f} {1:>6s}\n".format( # for total effect

100*(fixed_results[i][2]/fixed_results[0][2]), # Fixed weight

"----",

)

text += "{0:>5s}{1:>22.2f}\n".format( # for total effect

"----",

100*(random_results[i][2]/random_results[0][2])

)

continue

text +="{0:<18.2f} {1:>5.2f} \n".format(

100*(fixed_results[i][2]/fixed_results[0][2]), # Fixed weight

100*(random_results[i][2]/random_results[0][2])) # Random weight

return text

def totalsub(fixed_results,random_results): ##between-study variance

text="Fixed effect Model\n""Random effect Model:Tau\u00b2={0:.3f} I\u00b2={1:s} p-value={2:s}".format(

random_results[0][12],

str(round(random_results[0][9], 2)) + "%",

random_results[0][11])

return text

#create forest plot

def Fig_Forest (size,dpi,es_w_ci,fix_results, titletxt="Risk Ratio",no_ttl=False):

if es_w_ci[0][0] in "OR,RR" :

def _x_tran0(x):

return math.log(x)

def _x_tran1(x):

return math.exp(x)

elif es_w_ci[0][0] in "RD,MD,SMD" :

def _x_tran0(x):

return x

def _x_tran1(x):

return x

else:

Err="error.(failed to get effect size while drawing forest plot)"

raise Exception(Err)

myfig = plt.figure(linewidth=1, figsize=size, dpi=dpi) #Frameon=False, num="Forest plot by PythonMeta",

myfig.set_size_inches(size)

plt.title(titletxt)

ax = gca()

ax.spines['right'].set_color('none')

ax.spines['top'].set_color('none')

ax.spines['left'].set_color('none')

ax.xaxis.set_ticks_position('bottom')

ax.yaxis.set_ticks_position('left')

plt.xticks(fontsize=9)

plt.yticks(fontsize=9)

xlim=[];y_k=0;subgrp=[]

for i in range(len(es_w_ci)):

xlim.append(es_w_ci[i][3])

xlim.append(es_w_ci[i][4])

stdname=es_w_ci[i][0]

if stdname[0:5]=="<sub>": #this line is a subgroup

subgrp.append(es_w_ci[i])

xmin= _x_tran0(min(xlim))

xmax= _x_tran0(max(xlim))

xmax=max(abs(xmin),abs(xmax))

xmin=-xmax

plt.xlim(xmin*1.1,xmax*1.1)

ylabel=[i[0].replace("<sub>","") for i in es_w_ci[1:]]

if no_ttl==True :

ax.set_yticks(range(len(es_w_ci)))

ymax=len(es_w_ci)

ylabel.extend([""])

y_k=0

else:

ax.set_yticks(range(len(es_w_ci)+3))

ymax=len(es_w_ci)+3

ylabel.extend(["","Overall","",""])

y_k=3.5

plt.ylim(0, ymax)

ylabel.reverse()

ax.set_yticklabels(ylabel)

ax.set_xticklabels([round(_x_tran1(x),2) for x in ax.get_xticks()])

plt.plot([0,0], [0,len(es_w_ci)+3], 'black')

if len(subgrp)>0:

weight_all=subgrp[0][2]

else:

weight_all=es_w_ci[0][2]

N=es_w_ci[0][5];k=0;i_subgrp=0

for i in range(1,len(es_w_ci)):

stdname=es_w_ci[i][0]

if stdname[0:5]=="<sub>": #this line is a subgroup

i_subgrp+=1

if i_subgrp>len(subgrp)-1:

i_subgrp=len(subgrp)-1

weight_all=subgrp[i_subgrp][2]

x=[_x_tran0(es_w_ci[i][1]),

_x_tran0(es_w_ci[i][3]),

_x_tran0(es_w_ci[i][1]),

_x_tran0(es_w_ci[i][4]),

_x_tran0(es_w_ci[i][1])]

y=[len(es_w_ci)-i+y_k-0.6,

len(es_w_ci)-i+y_k-0.4,

len(es_w_ci)-i+y_k-0.2,

len(es_w_ci)-i+y_k-0.4,

len(es_w_ci)-i+y_k-0.6]

if (es_w_ci[i][9]<50) :

plt.fill(x,y,color="blue", lw=1) #filled: I2<50

else :

plt.plot(x,y, 'blue', lw=1) #empty: I2>50

x=[_x_tran0(fix_results[i][1]),

_x_tran0(fix_results[i][3]),

_x_tran0(fix_results[i][1]),

_x_tran0(fix_results[i][4]),

_x_tran0(fix_results[i][1])]

y=[len(fix_results)-i+y_k+0.5,

len(fix_results)-i+y_k+0.3,

len(fix_results)-i+y_k+0.1,

len(fix_results)-i+y_k+0.3,

len(fix_results)-i+y_k+0.5]

if (fix_results[i][9]<50) :

plt.fill(x,y,color="blue", lw=1) #filled: I2<50

else :

plt.plot(x,y, 'blue', lw=1) #empty: I2>50

continue

#weight

weight=es_w_ci[i][2]/weight_all

#shadow X line

lncolor,lnstyle=("blue","-")

plt.plot([_x_tran0(es_w_ci[i][3]),_x_tran0(es_w_ci[i][4])], [len(es_w_ci)-i+y_k,len(es_w_ci)-i+y_k], lncolor, linestyle=lnstyle, lw=0.6)

plt.plot((math.log(es_w_ci[0][1]),0),(math.log(es_w_ci[0][1]),(ymax+200)),linestyle="dotted", lw=0.5,color="k")

#central block

k=weight*0.2+0.05

x=[_x_tran0(es_w_ci[i][1])-k*(xmax*2.2/ymax),

_x_tran0(es_w_ci[i][1])+k*(xmax*2.2/ymax),

_x_tran0(es_w_ci[i][1])+k*(xmax*2.2/ymax),

_x_tran0(es_w_ci[i][1])-k*(xmax*2.2/ymax),

_x_tran0(es_w_ci[i][1])-k*(xmax*2.2/ymax)]

y=[len(es_w_ci)-i+y_k+k,

len(es_w_ci)-i+y_k+k,

len(es_w_ci)-i+y_k-k,

len(es_w_ci)-i+y_k-k,

len(es_w_ci)-i+y_k+k]

plt.fill(x,y,color=lncolor, lw=1) #filled:

if no_ttl==True:

pass

else:

#draw total ES from es_w_ci[0]

x=[_x_tran0(es_w_ci[0][1]),

_x_tran0(es_w_ci[0][3]),

_x_tran0(es_w_ci[0][1]),

_x_tran0(es_w_ci[0][4]),

_x_tran0(es_w_ci[0][1])]

y=[1.4,1.1,0.8,1.1,1.4]

if (es_w_ci[0][9]<50) :

plt.fill(x,y,color="black", lw=1) #filled: I2<50

else :

plt.plot(x,y, 'black', lw=1) #empty: I2>50

x=[_x_tran0(fix_results[0][1]),

_x_tran0(fix_results[0][3]),

_x_tran0(fix_results[0][1]),

_x_tran0(fix_results[0][4]),

_x_tran0(fix_results[0][1])]

y=[2.3,2,1.7,2,2.3]

if (es_w_ci[0][9]<50) :

plt.fill(x,y,color="black", lw=1) #filled: I2<50

else :

plt.plot(x,y, 'black', lw=1) #empty: I2>50

plt.xlabel("{:<38}{:<s}".format(" Favours Placebo","Favours Haloperidol"),fontsize=10)

return myfig

#df=numbers of datasets minus 1

#p-value for Q of subgroups

#wo=withoutmiss

#wm=with miss

#test for subgroup differences

def subQ(womiss_random,miss_random):

WY2wo=((math.log(womiss_random[0][1]))**2)*womiss_random[0][2]

WY2wm=((math.log(miss_random[0][1]))**2)*miss_random[0][2]

WY2=WY2wo+WY2wm

WYwo=(math.log(womiss_random[0][1]))*womiss_random[0][2]

WYwm=(math.log(miss_random[0][1]))*miss_random[0][2]

WYsq=(WYwo+WYwm)**2

W=womiss_random[0][2]+miss_random[0][2]

Q=round(WY2-(WYsq/W),3)

return Q

def pvalue(womiss_random,miss_random):

Qpv=(1-scipy.stats.chi2.cdf(subQ(womiss_random,miss_random),df=1))

Qp=round((Qpv),2)

return Qp

def main(setting):

d = PMA.Data() # Load Data class

m = PMA.Meta() # Load Meta class

f = PMA.Fig() # Load Fig class

d.datatype = setting["datatype"] # set data type, 'CATE' for binary data or 'CONT' for continuous data

studies = d.getdata(d.readfile("pythondata.txt")) # get data from a data file

withmiss=d.getdata(d.readfile("withmiss.txt"))

withoutmiss=d.getdata(d.readfile("withoutmiss.txt"))

m.subgroup = d.subgroup

m.datatype = d.datatype # set data type for meta-analysis calculating

m.models = setting["models"][0] # set effect models: 'Fixed'

m.algorithm = setting["algorithm"] # set algorithm, based on datatype and effect size

m.effect = setting["effect"] # set effect size:RR/OR/RD for binary data; SMD/MD for continuous data

results = m.meta(studies) # performing the analysis

m.models = setting["models"][1] #set effect models: Random"

res_random = m.meta(studies)

Fig_Forest((8,8),dpi=100,es_w_ci=(res_random),fix_results=(results))

plt.yticks([])

plt.text(10,2.8,showeight(results,res_random),fontsize=8,multialignment="left",linespacing=2.53,weight="bold")

plt.text(6,2.8,CI(results, res_random),fontsize=8,multialignment="left",linespacing=2.53,weight="bold")

plt.text(-9,12.4,ID(withmiss),multialignment="left",fontsize=8,linespacing=2.7)

plt.text(-9,4.2,ID(withoutmiss),multialignment="left",fontsize=8,linespacing=2.7)

plt.text(-9,11.2,rultswithmiss(results,res_random),multialignment="left",fontsize=8,linespacing=2,

weight="bold")

plt.text(-9,3,rultswithoutmiss(results,res_random),multialignment="left",fontsize=8,linespacing=2,

weight="bold")

plt.text(-9.5,6.3,"Without Missing Data",multialignment="left",fontsize=8,weight="bold",rotation=90)

plt.text(-9.5,16.5,"With Missing Data",multialignment="left",fontsize=8,weight="bold",rotation=90)

plt.text(-9,0.8,totalsub(results, res_random),multialignment="left",fontsize=8,weight="bold"

,linespacing=2.5)

plt.text(-6.5,12.4,showithmiss(withmiss),multialignment="left",fontsize=8,linespacing=2.7)

plt.text(-6.5,4.2,showithoutmiss(withoutmiss),multialignment="left",fontsize=8,linespacing=2.7)

plt.text(5.8,1,descrip(results,res_random),fontsize=8, style='normal', ha="left",

va='bottom',multialignment="left",linespacing=2,weight="bold")

#add subgroup difference. You should add it manually after extracting

plt.text(-9,0.1,"Test for subgroup difference(randomeffects) : Q=5.60 df=1 p=0.02",fontsize=8, style='normal', ha="left", va='bottom',multialignment="left",linespacing=2.5,weight="bold")

plt.savefig("subForest.png", dpi=300,bbox_inches="tight",orientation="landscape",transparent=True)

if __name__ == '__main__':

settings = {"datatype": "CATE", # for CATEgorical/count/binary/dichotomous data

"models": ["Fixed","Random"], # models: Fixed or Random

"algorithm": "MH", # algorithm: MH, Peto or IV

"effect": "RR"} # effect size: RR, OR, RD

main(settings)

**Figure 4. Comparison of summary Risk Ratios (RR) according to different missing data imputation methods**

**To create missing data imputation methods, according to description of each method we created dataset. We use the original Cochrane dataset with six variables. (** **resp.h, fail.h, drop.h, resp.p, fail.p ,drop.p). After creating the datasets, we ran a random-effect model, IV,DL on each dataset.**

**We used zEpid package to draw forest plots and compare the results.**

#ICA-0

import pandas as pd

import numpy as np

df= pd.read_csv("Cochrane.csv")

df.insert(df.columns.get_loc('fail.h'),"exptotal",df["resp.h"]+df["fail.h"]+df["drop.h"])

df.insert(df.columns.get_loc('fail.p'),"conttotal",df["resp.p"]+df["fail.p"]+df["drop.p"])

new_df=df.drop(["fail.h","drop.h","fail.p","drop.p"],axis=1)

new_df.to_csv(r'C:\Users\user\Desktop\ICA-0.csv',index = False)

#ICA-1

import pandas as pd

import numpy as np

df= pd.read_csv("Cochrane.csv")

df.insert(df.columns.get_loc('resp.h'),"resph",df["resp.h"]+df["drop.h"])

df.insert(df.columns.get_loc('resp.h'),"exptotal",df["resp.h"]+df["fail.h"]+df["drop.h"])

df.insert(df.columns.get_loc('resp.p'),"respp",df["resp.p"]+df["drop.p"])

df.insert(df.columns.get_loc('resp.p'),"conttotal",df["resp.p"]+df["fail.p"]+df["drop.p"])

new_df=df.drop(["resp.h","fail.h","drop.h","resp.p","fail.p","drop.p"],axis=1)

new_df.to_csv(r'C:\Users\user\Desktop\ICA-1.csv',index = False)

#ICA-WORST

import pandas as pd

import numpy as np

df= pd.read_csv("Cochrane.csv")

df.insert(df.columns.get_loc('resp.h'),"resph",df["resp.h"]+df["drop.h"])

df.insert(df.columns.get_loc('resp.h'),"exptotal",df["resp.h"]+df["fail.h"]+df["drop.h"])

df.insert(df.columns.get_loc('resp.p'),"respp",df["resp.p"]+df["drop.p"])

df.insert(df.columns.get_loc('resp.p'),"conttotal",df["resp.p"]+df["fail.p"]+df["drop.p"])

new_df=df.drop(["resp.h","fail.h","drop.h","resp.p","fail.p","drop.p"],axis=1)

new_df.to_csv(r'C:\Users\user\Desktop\ICA-1.csv',index = False)

#ICA-BEST

import pandas as pd

import numpy as np

df= pd.read_csv("Cochrane.csv")

df.insert(df.columns.get_loc('resp.h'),"resph",df["resp.h"]+df["drop.h"])

df.insert(df.columns.get_loc('resp.h'),"exptotal",df["resph"]+df["fail.h"])

df.insert(df.columns.get_loc('fail.p'),"conttotal",df["resp.p"]+df["fail.p"]+df["drop.p"])

new_df=df.drop(["resp.h","fail.h","drop.h","fail.p","drop.p"],axis=1)

new_df.to_csv(r'C:\Users\user\Desktop\ICA-best.csv',index = False)

#missing data imputation methods forest plot using zEpid package

#the results are from random effect model,IV,DL. Rechecked with R and STATA.

import matplotlib.image as mpimg

import numpy as np

import matplotlib.pyplot as plt

import zepid

from zepid.graphics import EffectMeasurePlot

labs = ["ACA(Isq=41.37% Tausq=0.146 pvalue=0.039 )",

"ICA0(Isq=25.78% Tausq=0.092 pvalue=0.16 )",

"ICA1(Isq=60.34% Tausq=0.121 pvalue=0.00 )",

"ICAb(Isq=25.94% Tausq=0.083 pvalue=0.16 )",

"ICAw(Isq=74.22% Tausq=0.465 pvalue=0.00 )"]

measure = [2.09,2.24,1.79,2.71,1.97]

lower = [1.49,1.63,1.33,2.00,1.25]

upper = [2.92,3.07,2.42,3.66,3.11]

p = EffectMeasurePlot(label=labs, effect_measure=measure, lcl=lower, ucl=upper)

p.labels(effectmeasure='RR')

p.colors(pointshape="D")

ax=p.plot(figsize=(7,3), t_adjuster=0.09, max_value=4, min_value=0.35 )

plt.title("Random Effect Model(Risk Ratio)",loc="right",x=1, y=1.045)

plt.suptitle("Missing Data Imputation Method",x=-0.1,y=0.98)

ax.set_xlabel("Favours Control Favours Haloperidol ", fontsize=10)

ax.spines['top'].set_visible(False)

ax.spines['right'].set_visible(False)

ax.spines['bottom'].set_visible(True)

ax.spines['left'].set_visible(False)

plt.savefig("Missing Data Imputation Method",bbox_inches='tight')

**Figure 5. Funnel plot of the 17 studies. The vertical line is the summary RR as estimated from the Fixed effect model**

import PythonMeta as PMA

import matplotlib.pyplot as plt

import math

from pylab import gca

from matplotlib.lines import Line2D

def logES(rults):

logES=[]

for i in range(1,len(rults)):

Yi=math.log(rults[i][1]) # effect size:rults[i][1]

logES.append(Yi)

return logES

#this is the standard error of the studies.In random effect models with DL method the standard error is the square root of the

# the variance and the variance is the reciprocal of the weights.So in random models we should use SE(rults)as the standard error

#it can be used in IV fixed model as well

def SE(rults):

SE=[]

for i in range(1,len(rults)):

VM=math.sqrt(1/rults[i][2])

SE.append(VM)

return SE

#to obtain the standard error in FIXED MH,IV method,we should use this function as standard error in the plot

def SEFix(rults):

SE=[]

for i in range(1,len(rults)):

se=rults[i][6]

SE.append(se)

return SE

#create funnel plot and use log effect size on the X axis

def funnelplot(rults):

fig=plt.figure(figsize=(9,8))

x=logES(rults)

y=SEFix(rults)

plt.plot(x,y,"o",lw=1)

plt.xlim(min(x)-4,max(x)+4) #the xlim can change manually

plt.ylim(max(y)+0.04,0)

ymax=max(y)+0.1

tes=(rults[0][1]) #total effect size

logtes=math.log(tes)

plt.plot ([logtes,logtes],[0,max(y)],color="blue", linestyle="--", lw=1)

plt.plot ([logtes,logtes-(1.96*max(y))],[0,max(y)],color="blue", linestyle="--", lw=1)

plt.plot ([logtes,logtes+(1.96*max(y))],[0,max(y)],color="blue", linestyle="--", lw=1)

ax = gca()

ax.spines['top'].set_visible(False)

ax.spines['right'].set_visible(False)

plt.ylabel("Standard Error",fontsize=14,rotation=90 )

plt.xlabel(" Favours Placebo logRR Favours Haloperidol",fontsize=10 )

def main(setting):

d = PMA.Data() # Load Data class

m = PMA.Meta() # Load Meta class

f = PMA.Fig() # Load Fig class

d.datatype = setting["datatype"]

studies = d.getdata(d.readfile("haloperidol.txt"))

m.datatype = d.datatype

m.models = setting["models"]

m.algorithm = setting["algorithm"]

m.effect = setting["effect"]

results = m.meta(studies)

funnelplot(results) #This is the funnel plot we created which seems more correct

#f.funnel(results) #This is the original funnel plot of the package

plt.savefig("Funnelplot.jpg",transparent=False,dpi=300)

if __name__ == '__main__':

settings = {"datatype": "CATE", # for CATEgorical/count/binary/dichotomous data

"models": "FIXED" , # models: Fixed or Random

"algorithm": "MH", # algorithm: MH, Peto or IV

"effect": "RR"} # effect size: RR, OR, RD

main(settings)

#Contour enhanced plot

def CEfunnelplot(rults): #to draw contour enhanced funnel plot

fig=plt.figure(figsize=(9,8))

ax=gca()

x=logES(rults)

y= SEFix(rults)

plt.xlim(min(x)-5,max(x)+2) #the xlim can change manually

plt.ylim(max(y)+0.1,0)

ax.set_facecolor(color="whitesmoke")

tes=(rults[0][1])

logtes=math.log(rults[0][1])

plt.plot ([0,(-2.58*max(y))],[0,max(y)], linestyle="None", lw=1)

plt.plot ([0,(2.58*max(y))],[0,max(y)], linestyle="None", lw=1)

trianglex = [ -2.58*max(y), 0, 2.58*max(y)]

triangley = [ max(y),0, max(y)]

for i in range(3):

plt.plot(trianglex, triangley,color="lightgray")

plt.fill(trianglex, triangley,"lightgray")

plt.plot ([0,(-1.96*max(y))],[0,max(y)], linestyle="None", lw=1)

plt.plot ([0,(1.96*max(y))],[0,max(y)], linestyle="None", lw=1)

trianglex = [ -1.96*max(y), 0,1.96*max(y)]

triangley = [ max(y),0, max(y)]

for i in range(3):

plt.plot(trianglex, triangley,color="darkgrey")

plt.fill(trianglex, triangley,"darkgrey")

plt.plot ([0,(-1.65*max(y))],[0,max(y)], linestyle="None", lw=1)

plt.plot ([0,(1.65*max(y))],[0,max(y)], linestyle="None", lw=1)

trianglex = [ -1.65*max(y), 0,1.65*max(y)]

triangley = [ max(y),0, max(y)]

for i in range(3):

plt.plot(trianglex, triangley,color="white")

plt.fill(trianglex, triangley,"white")

plt.plot ([logtes,logtes],[0,max(y)],color="blue", linestyle="--", lw=1)

plt.plot ([logtes,logtes-1.96*max(y)],[0,max(y)],color="blue", linestyle="--", lw=1)

plt.plot ([logtes,logtes+1.96*max(y)],[0,max(y)],color="blue", linestyle="--", lw=1)

plt.plot(x,y,"o",lw=1,color="k")

ax = gca()

ax.spines['top'].set_visible(False)

ax.spines['right'].set_visible(False)

colors = ['whitesmoke', 'lightgray', 'darkgrey',"white"]

lines = [Line2D([0], [0], color=c, linewidth=3, linestyle='None', marker="s") for c in colors]

labels = ["p < 1%","1%< p <5%","5%< p <10%","p > 10%"]

plt.legend(lines, labels,shadow=True)

plt.xlabel("logRR",fontsize=14 )

plt.ylabel("Standard Error",fontsize=14,rotation=90 )

plt.savefig("Contour Enhanced Funnel plot.jpg")

def main(setting):

d = PMA.Data()

m = PMA.Meta()

d.datatype = setting["datatype"]

studies = d.getdata(d.readfile("Haloperidol.txt"))

m.datatype = d.datatype

m.models = setting["models"]

m.algorithm = setting["algorithm"]

m.effect = setting["effect"]

results = m.meta(studies)

CEfunnelplot(results)

if __name__ == '__main__':

settings = {"datatype": "CATE", # for CATEgorical/count/binary/dichotomous data

"models": "FIXED" , # models: Fixed or Random

"algorithm": "MH", # algorithm: MH, Peto or IV

"effect": "RR"} # effect size: RR, OR, RD

main(settings)
